# NASAL CARRIAGE OF MULTI-DRUG RESISTANT PANTON VALENTINE LEUKOCIDIN POSITIVE *STAPHYLOCOCCUS AUREUS* IN HEALTHY INDIVIDUALS OF TUDUN-WADA, GOMBE STATE, NIGERIA

**DOI:** 10.21010/ajid.v15i1.3

**Published:** 2020-12-14

**Authors:** Adebola Onanuga, Ocholi Jonathan Adamu, Babatunde Odetoyin, Jabir Adamu Hamza

**Affiliations:** 1 Department of Pharmaceutical Microbiology & Biotechnology, Faculty of Pharmacy, Niger Delta University, Wilberforce Island, Bayelsa State, Nigeria; 2 Department of Pharmaceutical microbiology & Biotechnology, Faculty of Pharmaceutical Sciences, Gombe State University, Tudun-Wada, Gombe State, Nigeria; 3 Department of Medical Microbiology & Parasitology, Faculty of Basic Medical Sciences, Obafemi Awolowo University, Ile-Ife, Nigeria

**Keywords:** Nasal carriage, *Staphylococcus aureus*, MDR, PVL, *mecA* gene, healthy adults;

## Abstract

**Background:**

Panton-Valentine Leucocidin (PVL)-producing *Staphylococcus aureus* strains have been implicated in serious community-associated invasive infections and their increasing multidrug resistance is a major global health concern. Thus, we investigated the prevalence of the PVL gene and the antimicrobial resistance profile of nasal *S. aureus* isolates from healthy adults in Tundu-Wada, Gombe State of Nigeria.

**Methods and Materials:**

A total of 262 nasal samples from healthy adults were obtained and cultured. The isolates were identified as *S. aureus* by standard morphological and biochemical methods alongside with the Polymerase Chain Reaction (PCR) amplification of their *16S rRNA* gene. Antimicrobial susceptibility testing was performed by the disc diffusion technique and the presence of *mecA* and PVL genes was determined by PCR analysis.

**Results:**

The overall nasal colonization of *S. aureus* was 17.6%. The prevalence of haemolysin and biofilm production among the isolates was 25(54.3%) and 42(91.3%), respectively. Only 2(4.3%) and 5(10.9%) possessed *mecA* and PVL genes respectively but none of the isolates harboured these two genes. All the isolates were resistant to amoxicillin but were highly susceptible (93.7%) to gentamicin. The prevalence of multi-drug resistance (MDR) among the isolates was M 45.7% and all PVL-producing isolates were MDR while one of the isolates with *mecA* gene exhibited extensive-drug resistance (XDR).

**Conclusion:**

This is the first report of nasal colonization of MDR PVL-producing *S. aureus* in healthy adults in Gombe, Northeastern Nigeria. This study highlights the importance of routine surveillance of healthy populations to provide useful strategies for controlling the spread of virulent multidrug-resistant organisms within the community.

## Introduction

*Staphylococcus aureus* is an opportunistic pathogen that colonizes the anterior nares, vagina and skin of the healthy human populations (Eibach *et al.*, 2017). The anterior nares are the best ecological habitat of *S. aureus* and 20 to 30% of the general human populations are *S. aureus* nasal carriers while one-third of them are persistently colonized (Kaspar *et al.*, 2016).

*S. aureus* produces a wide variety of virulence factors which include a spectrum of exotoxins like alpha-haemolytic toxin, Panton-Valentine leukocidin (PVL) and biofilms that contribute to its ability to colonize, invade and evade host immunological responses and also form protective barriers to several antibiotics (Archer *et al.*, 2011). *S. aureus* is therefore a major cause of serious infections ranging from skin abscesses to life-threatening infections in both clinical and community settings worldwide (Shittu *et al.*, 2011). Nasal *S. aureus* colonization has been identified as the origin of invasive infections in its carriers and the healthy carriers are subsequent sources of transmission of these infections in the communities (Asiimwe *et al.*, 2017).

Methicillin-Resistant *Staphylococcus aureus* (MRSA) strains are a major nosocomial pathogen with reduced susceptibility to different antibacterial classes. They cause a wide range of severe infections from soft tissue infections to life-threatening toxic shock syndromes in the hospitals worldwide. Methicillin resistance in *S. aureus* is conferred by *mecA* gene that encodes a modified Penicillin Binding Protein 2a (PBP2a) with a low affinity for beta-lactam antibiotics and conferment of cross-resistance to some other classes of antimicrobial agents. Treatment of *S. aureus* infections has faced severe difficulties due to the global spread of MRSA strains (Shittu *et al.*, 2011).

The increasing worldwide emergence of MRSA infections in community settings has become a major public health concern since they can occur in healthy individuals lacking any health care-associated exposure (Aqel *et al.*, 2015; Asiimwe *et al.*, 2017). The community-associated MRSA (CA-MRSA) strains have been identified to be more virulent and exhibit multi-drug resistance than those isolated from hospital settings (Bettin *et al.*, 2012). Also, about 30% of *S aureus* infections have been attributed to CA-MRSA strains and their infections are majorly superficial skin and soft-tissue infections which can easily be spread by human to human contact. The production of Panton-Valentine Leukocidin (PVL) has been described as the prominent feature of CA-MRSA (van der Meeren *et al.*, 2014).

Panton-Valentine leukocidin (PVL) is a pore-forming cytotoxin bicomponent that is expressed by some strains of *S. aureus*. This toxin was named in 1932 after two scientists Sir Philip Noel Panton and Francis Valentine who associated it with soft tissue infections caused by a member of the family synergohymenotropic toxin that induces pores in the membrane cells (Bhatta *et al.*, 2016). PVL forms pores in the membrane of host defense cells by targeting polymorphonuclear cells, monocytes, and macrophages through synergistic action of two secretory proteins LukS-PV and LukF-PV, which are encoded by two co-transcribed genes of a prophage integrated in the *S. aureus* chromosome (Genestier *et al.*, 2005; Yoong and Torres, 2013). PVL production in *S. aureus* strains has been associated with an array of diseases; from mild skin and soft tissue infections to more fatal, like haemorrhagic necrotizing pneumonia in healthy young adults (Hu *et al.*, 2015). PVL genes are mostly associated with CA-MRSA infections (Shittu *et al.*, 2011).

However, recent studies have also described the presence of PVL genes in HA-MRSA strains, a situation that can result into the emergence of multidrug-resistant HA-MRSA strains with increased virulence capacity (Omuse *et al.*, 2013; Hu *et al.*, 2015).

This study therefore aimed at determining the asymptomatic carrier rate of PVL genes and antimicrobial resistance characteristics of nasal *S. aureus* isolated from healthy inhabitants of Tudun-Wada, Gombe State, as a means of providing the first information on the surveillance of *S. aureus* colonization of healthy humans in this part of North-East Nigeria.

## Materials and Methods

### Study Population

The apparently healthy inhabitants of Tundun Wada in the city of Gombe, Gombe State, who had not taken a course of antimicrobial therapy, nor been admitted to a hospital within a period of three months prior to the commencement of the survey, were recruited into this cross-sectional study from April to July, 2018. Tundun Wada is a popular town in Gombe Local Government, Gombe State in the North-Eastern part of Nigeria where the Gombe State University is located and it is situated on latitude 11.2335° North, longitude 11.0955° East. The major occupations of its inhabitants are civil service, trading and schooling.

### Study Design and Settings

A cross-sectional study aimed at determining the prevalence rate of nasal *S. aureus* and its antibiotic characteristics was carried out randomly among 262 healthy inhabitants of Gombe metropolis as a representative of healthy adults in the community. The sample size was determined using the average prevalence of nasal *S. aureus* among similar subjects in the previous studies (Charan and Biswas, 2013). The volunteers were randomly enrolled from the University’s lecture rooms and its neighbouring community.

### Ethical Clearance

Ethical approval was obtained from the Ethics and Research committee of Gombe State University, Gombe, Nigeria (GSU/FPHARS/PMB/V.1/015 on 12/02/2018). This facilitated the commencement of the study in April 2018. The volunteers gave written informed consent and filled questionnaire to supply their basic demographic data, like gender and age, with the exclusion of their names and addresses before the collection of nasal specimens.

### Sample collection and Bacteriology

Nasal specimens were collected from each of the individual volunteers with sterile swab sticks moistened with sterile normal saline solution (0.9%^w^/_v_ Sodium chloride) and transported immediately to the Department of Pharmaceutical Microbiology and Biotechnology laboratory for inoculation and streaking on the already prepared sterilized Nutrient Agar plates. The discrete colonies obtained from the inoculated Nutrient agar (Oxoids, UK) plates after incubation at 37^o^C for 24 h were sub-cultured on Mannitol Salt Agar (Oxoids, UK) and Chromogenic agar (Difco™ orientation) plates and incubated at 35^o^C for 24-48 h. The colonies that were characteristic of *S aureus* on Mannitol Salt agar and Chromogenic agar plates were further identified using a combination of standard microbiological methods and biochemical tests which include colonial morphology, Gram’s stain reaction, catalase, coagulase, mannitol fermentation and DNase tests.

### Preparation of DNA template for PCR Amplification

The DNA templates of each of the biochemically confirmed *S. aureus* isolates were generated by suspending much of the overnight culture’s colonies of each of the isolates on Nutrient agar plates into 100 µL 1X Tris-EDTA buffer, vortexed and boiled at 100°C for 10 minutes as described by Onanuga *et al*. (2014). The boilate was transferred immediately to the freezer (-20°C) for 10 minutes, maintained at room temperature, vortexed and centrifuged at 10, 000 rpm for 10 minutes. The resulting supernatant containing DNA of each isolate was collected, stored at 4°C and used as a DNA template for PCR analyses.

### Molecular Identification of *S. aureus* through PCR amplification of *16SrRNA* gene

The isolates that were Gram-positive grape-like clustered cocci, positive to catalase test, mannitol fermentation test, DNase test, coagulase tests were subjected to PCR analysis for the presence of *S. aureus* *16SrRNA* gene as a confirmation for *S. aureus*.

The *S. aureus 16SrRNA* gene was screened for in each of the isolate using the primers described by Matsuda *et al*. (2007). The *S. aureus* specific *16SrRNA* gene was amplified using a 21-nucleotide forward primer 5’-ACGGTCTTGCTGTCACTTATA-3’ and 24-nucleotide reverse primer 5’-TACACATATGTTCTTCCCTAATAA-3’ with a product size of 257 bp. The PCR was carried out in a thermal cycler using a 25µl reaction mixture containing 12.5 µl 2x master mix, 0.5 µl each of the forward and reverse primers, 8.5 µl of nuclease-free water and 3 µl of the DNA template at the following conditions: initial denaturation at 94^o^C for 4 mins, 37cycles of 94^o^C for 1min, 50^o^C for 30 sec and 72^o^C for 1min and a final elongation at 72^0^ for 5mins. The amplified PCR products (10 µl) were evaluated on a 1.5% (w/v) agarose gel at 100 mV for 60 min using BIO-RAD Power Pac 3000 with a molecular weight marker (100 bp DNA Ladder). The DNA bands were then visualized and photographed under UV light after staining the gel with ethidium bromide.

### The Detection of *PVL* and *MecA* genes in the *S. aureus* isolates

The *S. aureus* isolates with the specific *16SrRNA* gene were screened for the presence of *Panton Valentine Leukocidin* (*PVL*) and Methicillin resistance (*MecA*) genes using the primers described by Kaur *et al*. (2012) and Shittu *et al*. (2006) respectively. The *PVL* gene was amplified using a 31-nucleotide forward primer 5’-ATCATTAGGTAAAATGTCTGGACATGATCCA-3’ and 27-nucleotide reverse primer 5’-GCATCAAGTGTATTGGATAGCAAAAGC-3’ with a product size of 433 bp while the *mecA* gene was amplified using a 20-nucleotide forward primer 5’-CTCAGGTACTGCTATCCACC-3’ and 19-nucleotide reverse primer 5’-CACTTGGTATATCTTCACC-3’ with a product size of 449 bp. The PCR reactions for PVL and *MecA* were carried out in a thermal cycler using a 25µl reaction mixture containing 12.5 µl 2x master mix, 0.5 µl each of the forward and reverse primers, 8.5 µl of nuclease-free water and 3 µl of the DNA template at the following conditions; initial denaturation at 94^o^C for 5 mins, 37cycles of 94^o^C for 1 min, 55^o^C for 30 sec and 72^o^C for 1min and a final elongation at 72^0^ for 7mins. The amplified PCR products (10 µl) were evaluated on a 1.5% (^w^/_v_) agarose gel at 100 mV for 60 min using BIO-RAD Power Pac 3000 with a molecular weight marker (100 bp DNA Ladder). The DNA bands were then visualized and photographed under UV light after staining the gel with ethidium bromide.

### Screening of *S. aureus* isolates for Haemolysin production

Haemolytic property of the *S. aureus* isolates was determined by inoculating the isolates on freshly prepared sterile 5%^v^/_v_ Blood Agar (consisting of 5 ml of human blood in 100 ml of Nutrient Agar) plates using straight wire loop and incubated at 37°C for 24 hours. Thereafter, plates were observed for green to black colouration of the agar (partial lysis of red blood cells – alpha haemolysis) and clear zones (complete lysis of red blood cells – beta haemolysis) around inoculated organisms indicating the production of haemolysin.

### Screening of *S. aureus* isolates for Biofilm production

Biofilm production in *S. aureus* isolates was performed using Congo Red Agar medium which was prepared using the combination of brain heart infusion agar 52 g/L, sucrose 50 g/L and Congo red indicator 8 g/L as described by (Mathur *et al.*, 2006). The Congo red was prepared as concentrated aqueous solution separately from other medium constituents and then sterilized in different containers before adding them together when the agar had cooled to 55°C before distributing to sterile plates to solidify. The plates were then inoculated with the test organisms and incubated at 37°C for 24 hours before examining for black colonies with a dry crystalline consistency indicating biofilm production.

### Antimicrobial Susceptibility Testing of *S. aureus* isolates

The antimicrobial susceptibility of the *S. aureus* to amoxicillin/clavulanic acid (20/10 μg), Linezolid (30 μg), sulphamethoxazole/trimethoprim (1.25/23.75 μg), gentamicin (10 μg), cefoxitin (30 μg), ciprofloxacin (5 μg), amoxicillin (30 μg), vancomycin (30ug), doxycycline (30ug) and erythromycin (15 μg) was determined by employing the modified Kirby-Bauer disc diffusion technique in accordance with the Clinical and Laboratory Standard Institute guidelines (CLSI, 2017). The *S. aureus* isolates that were resistant to at least one agent in three or more of the nine classes of antimicrobial agents used in this study were defined as having multi-drug resistance (MDR) (Magiorakos *et al.*, 2012).

### Statistical analysis

The groups’ differences were tested using the Chi-square test (or Fisher’s exact test when expected frequencies were too low), with the assumed level of statistical significance at a P-value of < 0.05. Data analysis was performed with SPSS version 15.0 for Windows (SPSS Inc, USA).

## Results

### Study Population

A total of 262 nasal swab samples were obtained from apparently healthy volunteers comprising 132 (50.4%) University students and 130 (49.6%) non-students inhabiting Tundun wada area of Gombe State. The volunteers were 90 (34.4%) females and 172 (65.6%) males of age range 15-62 years with an average age of 25.1 years as shown in Table 1.

**Table 1:** Age and gender distribution of the volunteers

Age groups	No. of samples	Female (%)		Male (%)	

		Students	Non-students	Students	Non students
15-20	70	26	9	20	15
21-26	118	27	11	44	36
27-32	39	4	4	9	22
33-38	23	0	4	2	17
39-44	3	0	1	0	2
45-50	2	0	1	0	1
51-56	3	0	1	0	2
57-62	4	0	2	0	2

**TOTAL**	**262**	**57 (21.8)**	**33 (12.6)**	**75 (28.6)**	**97 (37.0)**

### The Prevalence of *S. aureus* isolates among the healthy volunteers

**Figure 1:**
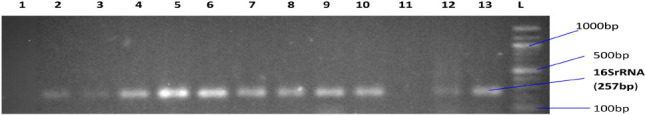
Agarose gel electrophoresis showing the amplified 16S1RNA gene of isolates Lanes 2, 3, 4, 5, 6, 7, 8, 9, 10, 12 and 13 showing positive bands for 16SrRNA gene (257bp) while 1 and 11 are negative. Lane L represents the lOObn Ladder.

The volunteers’ nasal samples yielded a total of 54 (20.6%) *S. aureus* isolates using conventional biochemical tests. However, only 46 (85.2%) of these isolates possessed the *S. aureus* gene-specific *16SrRNA* thereby confirming only 46 (17.6%) as the nasal carriage rate of *S. aureus* among the volunteers in this study (Figure 1). The observed differences in the carriage rates of *S. aureus* among the two groups of categories (*P >* 0.05) as shown in Table 2, are not statistically significant.

**Table 2:** Prevalence of *S. aureus* among the volunteers

Subjects	Sample number	No.(%) of *S. aureus*	P-value
**Status**			
Students	132	24 (18.2)	0.789
Non students	130	22 (16.9)	
**Gender**			
Female	90	19 (21.1)	0.274
Male	172	27 (15.7)	

**Total**	**262**	**46 (17.6)**	

### Virulence Characteristics of volunteers’ *S. aureus* isolates

In all the confirmed *S. aureus* samples, 25 (54.3%) and 42 (91.3%) phenotypically produced haemolysin and biofilm respectively. All, except two of the haemolysin producing isolates produced biofilm whilst only two of the total isolates lack these traits. The differences of these virulence traits observed among the gender and volunteers’ categorical status were not statistically significant (*P* > 0.05) except in the case of haemolysin production where more isolates of *S. aureus* with this ability were significantly recovered from males compared to their female counterparts (*P* = 0.009) Table 3.

**Table 3:** Prevalence of virulent *S. aureus* among the volunteers

Subjects	No. of isolates	PVL (%)	P-value	Haemolysin (%)	P-value	Biofilm (%)	P-value
**Status**							
Students	24	4 (16.7)	0.349^a^	14 (58.3)	0.571^b^	23 (95.8)	0.336^a^
Non-students	22	1 (4.5)		11 (50.0)		19 (86.4)		
**Gender**							
Female	19	2 (10.5)	1.000^a^	6 (31.6)	0.009^b*^	19 (100)	0.131^a^
Male	27	3 (11.1)		19 (70.4)		23 (85.2)		
**TOTAL**	46	5 (10.9)	25 (54.3)	42 (91.3)	

Key:

a Fisher Exact test

b Chi-Square test

* Statistically significant (p< 0.05).

**Figure 2:**
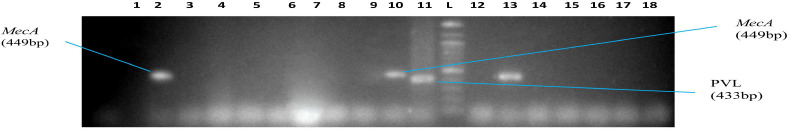
Agarose gel electrophoresis showing the amplified MccA and PVL genes of isolates Lanes 2 and 10 showing positive bands for MecA gene (449 bp) and Lanes 1 1 and 13 showing positive bands for PVL gene (433bp). Lane L represents the 1 OObp Quick-Load Molecular ladder.

The screening for the presence of PVL gene (Figure 2) among the isolates revealed a prevalence rate of 5 (10.9%), which were all biofilm producers except 3 that also produced haemolysin. The observed differences among the studied categories are not significant (*P >* 0.05) Table 3.

### Antimicrobial susceptibility patterns of *S. aureus* and MRSA isolates

**Figure 3:**
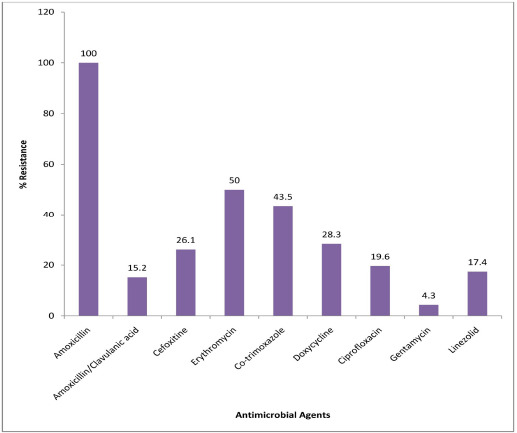
Antimicrobial resistance pattern of S. *aureus* isolates

The antimicrobial susceptibility testing revealed that *S. aureus* isolates had total resistance to amoxicillin, moderate resistance to erythromycin (50%) and co-trimoxazole (43.5%), but lower resistance (4–29%) to amoxicillin/clavulanic acid, ciprofloxacin, gentamicin, doxycycline, linezolid and cefoxitin. The most effective agent in this study was gentamicin to which the isolates exhibited the least resistance (4.3%) as shown in Figure 3.

The phenotypic estimation of methicillin resistance among the isolates in accordance with Clinical and Laboratory Standard Institute (CLSI) guidelines using resistance to cefoxitin revealed 26.1%. However, only two (2/12; 16.7%) of these cefoxitin-resistant *S. aureus* possessed *mecA* gene using PCR analysis (Figure 2).

### Prevalence of Multiple Drug Resistance among the Isolates

The determination of multiple antimicrobial resistance among the *S. aureus* isolates from the healthy volunteers revealed that only one (2.2%) isolate was fully susceptible to the eight classes of antimicrobials used whilst 21 (45.7%) isolates were multi-drug resistant. Among the MDR isolates, 20 (95.2%) phenotypically expressed biofilm production while 13 (61.9%) expressed haemolysin production. All the isolates that harboured the *PVL* gene were MDR whereas only one (50%) of the isolates with *mecA* gene was MDR. As shown in Table 4, all the MDR isolates exhibited 17 types of antibiogram and the most predominant combinations were “AML, SXT, E” (11, 52.4%), “AML, SXT, DO” (8, 38.1%) and “AML, CIP, SXT, E” (7, 33.3%).

**Table 4:** Antibiogram of multi-drug resistant *Staphylococcus aureus*

Number of classes of antimicrobials	Multidrug Resistance Pattern	Number of resistant isolates
3	AML, SXT, DO	1
3	AML, CN, SXT	1
3	AML, SXT, E,	2
3	AML, DO, FOX	1
3	AML, SXT, LZD	1
3	AML, E, DO	1
4	AML, CIP, SXT, DO	1
4	AML, E, LZD, FOX,	2
4	AML, CIP, SXT, E	1
4	AML, SXT, DO, LZD	1
5	AML, CIP, SXT, E, FOX	1
5	AML, CIP, SXT, DO, FOX	1
5	AML, SXT, E, LZD, FOX	2
5	AML, CIP, SXT, E, DO,	1
6	AML, CIP, SXT, E, FOX, CN	1
6	AML, CIP, SXT, E, DO, FOX	2
7	AML, CIP, SXT, E, DO, LZD, FOX	1

Total MDR (≥ 3)		21 (45.7%)

Symbols: AML- Amoxicillin, CIP- Ciprofloxacin, CN- Gentamicin, E- Erythromycin, FOX- Cefoxitin, SXT- Cotrimoxazole, DO- Doxycline and LZD- Linezolid

## Discussion

Nasal *S. aureus* colonization has been identified as the source of many invasive infections that are easily transmissible in the communities by apparently healthy carriers (Asiimwe *et al.*, 2017). However, the increasing emergence of PVL positive multidrug-resistant MRSA infections among individuals in the communities that lack any hospital-associated exposure is becoming a serious public health problem. This challenges the clinicians in the area of effective treatment (Bettin *et al.*, 2012; Aqel *et al.*, 2015; Asiimwe *et al.*, 2017).

Our study which is the first nasal *S. aureus* colonization surveillance at the Gombe metropolis, to the best of our knowledge, revealed the *S. aureus* gene-specific confirmed carrier rate of 17.6% with no significant differences (P> 0.05) among the genders and students/non-students categories of the healthy volunteers, suggesting that no gender or occupation status predisposes to nasal *S. aureus* colonization as reported by Adesida *et al. (*2007). This carrier rate is a little lower than the widely reported rate among asymptomatic nasal carriers (20-30%) (Weidenmaier *et al.*, 2012; Ellis *et al.*, 2014) but it is similar to the report of Adesida *et al*. (2007) in Lagos, Nigeria (14%); and Omuse *et al*. (2012) in Kenya (18.3%). It is however in contrast with the reports of AL-Haj *et al*. (2017) in Yemen (23.1%) (Ayepola *et al.*, 2018). In Ogun, Nigeria (56.7%) and (Nsofor *et al.*, 2015) in Owerri, Nigeria (56.3%) which revealed very high carrier rates among their healthy subjects.[U1] The observed differences might mainly be due to the screening techniques, geographical location and characteristics of the study population. This study also revealed that confirmation of *S. aureus* by conventional biochemical tests without specific gene probing could give 85.2% (46/52) specificity with a higher false carrier rate (a 20.6% carrier rate using only conventional biochemical methods was observed in this study).

The phenotypic screening for haemolysin production among the isolates revealed a prevalence rate of 54.3% in which the male group had a significantly higher prevalence (41.3%) than their female counterpart (13.0%) (*P*=0.009) while the observed difference among the students/non-students category was not significant (*P*=0.571). The moderately high prevalence of these nasal *S. aureus* strains, although lower than that reported by Onanuga *et al*. (2019) (78.7%) among asymptomatic University students in Bayelsa State, suggests the possibility of the development of opportunistic infections by the carriers since the strains with alpha-haemolytic toxin have been associated with significant disease burden in healthy humans (Berube and Wardenburg., 2013). The significantly higher prevalence observed among the male group could be a pointer to the marked difference in the personal hygiene status of the genders in the study area, suggesting that poor personal hygiene practice could predispose an individual more to the carriage of virulent organisms which can be easily disseminated to a vulnerable healthy community.

A high proportion (91.3%) of isolates from this study expressed biofilm production phenotypically suggesting the likelihood of a very serious public health problem in this study area. Organisms with biofilm production have been reported to exhibit varying levels of resistance to antimicrobial agents and host defense strategies, causing infections that are non-responsive to treatments (Archer *et al.*, 2011; de la Fuente-Nunez *et al.*, 2013). Our observed biofilm production prevalence is higher than the previous study in Bayelsa State, Nigeria (63.8%) (Onanuga *et al.*, 2019) and in Nepal (61%) (Devapriya *et al.*, 2014) from healthy individuals using the same screening technique. Hence, the behavioural use of anti-infective agents by the study populations in the various centres which in turn dictate the organism’s responsive strategies might be the reason for the observed differences.

The Panton-Valentine Leukocidin (PVL) gene encoding cytotoxin production in some strains of *S. aureus* which have been implicated in the community-associated invasive infections like skin and soft tissue infections (Hu *et al.*, 2015), was detected in five (10.9%) isolates. Our finding is similar to the report by Rebollo-Pérez *et al*. (2011) in which 15% of the nasal *S. aureus* from healthy preschool children were positive to PVL. In contrast to this finding is the report of a very high prevalence of 10 (62.5%) positive PVL among 16 nasal *S. aureus* from healthy children of Southwestern China by Gong *et al*. (2017). Further reports of PVL positive nasal *S. aureus* from healthy hospital staff carriers showed a prevalence of 24.4% in Kenya (Omuse *et al.*, 2013) among the isolated *S. aureus*. Reports of PVL positive nasal *S. aureus* from healthy individuals are however scarce in Nigeria. The prevalence of PVL positive *S. aureus* in our study is however low (1.91%; 5/262) among the screened volunteers but they are potential carriers of these strains that cause serious invasive infections which can be easily transmitted through hand contact and spread to other individuals in the community except the practice of proper personal hand hygiene is enforced in this area.

The studied isolates had moderate to total resistance (43-100%) to erythromycin, co-trimoxazole and amoxicillin, the antibiotics that are commonly used for community-associated *S. aureus* infections like skin and soft tissue infections. However, lower levels of resistance (4-29%) were exhibited by the isolates to other tested antibiotics with gentamicin having the least resistance (4.3%). Antibiotic resistance of Nasal *S. aureus* isolates from apparently healthy individuals has been widely reported in many quarters of Nigeria and other developing countries of the world with varying degrees of resistance (Conceicao *et al.*, 2014; Nsofor, 2015; Ayepola *et al.*, 2018). Antibiotic selection pressure within the communities and animal husbandry coupled with lack of enforcement of regulations regarding the sale and use of antibiotics in our environments have been associated with the increasing rate of this organism’s resistance to commonly used anti-infective agents, thereby making the treatment of this organism’s common community associated infections increasingly difficult (Shittu *et al.*, 2011).

The prevalence of methicillin resistance among the studied nasal *S. aureus* isolates (MRSA) measured by the organism resistance to cefoxitin was found to be 26.1% (12/46) but the expression of *mecA* was only detected in two (16.7%) of this phenotypic MRSA, indicating that only 2 (4.3%) of the nasal *S. aureus* isolates in this study possessed the *mecA* gene. This observation has been reported in many studies in Iran, Australia and China where only 30%, 24% and 12% of their studied MRSA isolates possessed the *mecA* gene respectively (Li *et al.*, 2001; Turnidge *et al.*, 2007; Motamedi *et al.*, 2015). This therefore explains the possibility of other mechanisms beside the *mecA* gene for methicillin resistance such as the *mecC* gene which has also been reported to encode for methicillin resistance in *S. aureus* but was not investigated in this study (Paterson *et al.*, 2014). Several studies have reported detection of the *mecA* gene in all their studied phenotypic MRSA isolates but strains of MRSA with the *mecA* gene were first discovered in the hospital-associated nosocomial infections that are characterized by multiple drug resistance to other non beta-lactams antibiotics before their detection in the community. This implies that these hospital strains have been transmitted to the community by the hospital staff and visitors (Rebollo-Pérez *et al.*, 2011; Aqel *et al.*, 2015; Gong *et al.*, 2017). Hence, the only two strains of MRSA with *mecA* gene detected among our studied apparently healthy adults (0.8%; 2/262) might have their source from the hospital settings which might cause HA-MRSA infections in the carriers and thus exposing the vulnerable population to the infections upon subsequent transmission. However, the analysis of the staphylococcal cassette chromosome mec (SCCmec) elements that will characterize the isolates and confirm our postulation is beyond the scope of this study.

The presence of the Panton-Valentine Leukocidin (PVL) gene among strains of *S. aureus* has been identified as a pathogenicity marker for community-associated infections and its co-occurrence with *mecA* gene in MRSA strains has been implicated in the serious community associated infections with increased morbidity and mortality (Rebollo-Pérez *et al.*, 2011). In this study, the five isolates with PVL gene were distributed among three methicillin-susceptible *S. aureus* (MSSA) and two MRSA that lack the *mecA* gene, which suggests that none of the studied isolates has the capacity to cause such serious infections that are characterized with increased morbidity and mortality in the community. This observation of PVL positive *S. aureus* isolates that lack the *mecA* gene has been reported in hospitals specimens in Southwestern Nigeria (Alli *et al.*, 2012) and Ahvaz, Iran (Motamedi *et al.*, 2015).

The estimation of multiple drug resistance among the isolates revealed that 21 (45.7%) of the studied isolates were multi-drug resistant (MDR) having “AML, SXT, E” (11, 52.4%) as the most prominent antibiogram and 20 (95.2%) of these MDR isolates phenotypically expressed biofilm production while 13 (61.9%) of them expressed haemolysin production. This observation further suggests that biofilm production in bacteria is a strong risk factor for multiple antimicrobial resistance and virulence capability (Archer *et al.*, 2011; de la Fuente-Nunez *et al.*, 2013). The prevalence of MDR in this study is lower than that reported by Onanuga *et al*. (2019) in Amassoma, South southern Nigeria (87.4%) from similar subjects suggesting that the individuals in this area might not be largely exposed to antimicrobials like those in the South southern Nigeria. Also, all the isolates with the PVL gene were MDR while one of the two MRSA strains with *mecA* gene was found to be extensively drug-resistant (XDR) as described by Magiorakos *et al*. (2012) having only been susceptible to one category of antimicrobials while the remaining one was a non-MDR. This observation suggests that the XDR-MRSA strain could be of the hospital setting while the non-MDR MRSA might be associated with the community setting since MRSA strains from hospital settings are characterized by high multiple drug resistance (Alli *et al.*, 2012; Aqel *et al.*, 2015; Gong *et al.*, 2017). However, the observed multi-drug resistance in all the isolates with PVL gene suggests that the increasing indiscriminate and non-regulatory use of antimicrobials in the community might be a significant risk factor for community-associated infections with very limited treatment options, a situation that calls for strategies to prevent the irrational use of antimicrobials and training on the proper personal hand hygiene in order to contain the spread of these MDR strains to other communities hosting the vulnerable healthy populations in our society.

## Conclusions

Our study revealed the carriage of multi-drug resistant Panton valentine leukocidin positive nasal *S. aureus* and extensively drug-resistant MRSA among apparently healthy inhabitants in Gombe environs, Northeastern Nigeria. This is the first surveillance report of the nasal colonization of *S. aureus* in this environment. These findings therefore emphasize the usefulness of routine epidemiological surveillance of healthy human populations as a means of developing strategies for the prevention of difficult-to-treat disease outbreaks in the community.

**List of abbreviations:**
PVL: - Panton-Valentine Leucocidin
PCR: - Polymerase Chain Reaction
MDR: - Multi-drug resistance
XDR: - extensively-drug resistance
AML: - Amoxicillin
CIP: - Ciprofloxacin
CN: - Gentamicin
E: - Erythromycin
FOX: - Cefoxitin
SXT: - Co-trimoxazole
DO: - Doxycline
LZD: - Linezolid
MRSA: - Methicillin-Resistant *Staphylococcus aureus*
PBP: - Penicillin Binding Protein
CLSI: - Clinical and Laboratory Standard Institute
